# Fitness cost of insecticide resistance on the life-traits of a *Anopheles coluzzii* population from the city of Yaoundé, Cameroon

**DOI:** 10.12688/wellcomeopenres.16039.2

**Published:** 2020-09-08

**Authors:** Diane Leslie Nkahe, Edmond Kopya, Borel Djiappi-Tchamen, Wilson Toussile, Nadege Sonhafouo-Chiana, Sevilor Kekeunou, Remy Mimpfoundi, Parfait Awono-Ambene, Charles Sinclair Wondji, Christophe Antonio-Nkondjio

**Affiliations:** 1 Malaria Research Laboratory, OCEAC, Yaoundé, Centre, PO Box 288, Cameroon; 2 Department of Animal Biology, University of Yaoundé 1, Yaoundé, Cameroon; 3 Department of Animal Biology, University of Dschang, Dschang, Cameroon; 4 Polytechnique, University of Yaoundé 1, Yaoundé, Cameroon; 5 Faculty of Sciences, University of Buea, Buea, Cameroon; 6 Department of Vector Biology, Liverpool School of Tropical Medicine, Liverpool, UK

**Keywords:** life-traits, An. coluzzii, insecticide resistance, fitness cost, Yaoundé, Cameroon

## Abstract

**Background:** Pyrethroid resistance is rapidly expanding in *An. gambiae* s.l. populations across Sub-Saharan Africa. Yet there is still not enough information on the fitness cost of insecticide resistance . In the present study, the fitness cost of insecticide resistance on *Anopheles coluzzii* population from the city of Yaoundé was investigated.

**Methods:** A resistant *An. coluzzii *colony was established from field collected mosquitoes resistant to both DDT and pyrethroid and selected for 12 generations with deltamethrin 0.05%. The Ngousso laboratory susceptible strain was used as control. A total of 100 females of each strain were blood fed and allowed for individual eggs laying, and then different life traits parameters such as fecundity, fertility, larval development time, emergence rate and longevity were measured. The TaqMan assay was used to screen for the presence of the L1014F and L1014S *kdr* mutations.

**Results:**  Field collected mosquitoes from the F0 generation had a mortality rate of 2.05% for DDT, 34.16% for permethrin and 50.23% for deltamethrin. The mortality rate of the F12 generation was 30.48% for deltamethrin, 1.25% for permethrin  and 0% for DDT. The number of eggs laid per female was lower in the resistant colony compared to the susceptible (p <0.0001). Insecticide resistant larvae were found with a significantly long larval development time (10.61±0.33 days) compare to susceptible (7.57±0.35 days). The number of emerging females was significantly high in the susceptible group compared to the resistant . The adults lifespan was also significantly high for susceptible (21.73±1.19 days) compared to resistant (14.63±0.68 days). Only the L1014F- *kdr* allele was detected in resistant population..

**Conclusion:** The study suggests that pyrethroid resistance is likely associated with a high fitness cost on *An.coluzzii* populations. The addition of new tools targeting specifically larval stages could improve malaria vectors control and insecticide resistance management.

## Introduction

Malaria prevention mainly relies on the use of vector control measures with indoor residual spraying (IRS) and long-lasting insecticidal nets (LLINs) as the core interventions ^1^. Five insecticide families, organophosphates, organochlorines, carbamates, pyrethroids and neonicotinoid are used in public health ^2^. The massive use of insecticides particularly pyrethroids over the last decades in vector control and in agriculture resulted in rapid expansion of insecticide resistance, which now affects almost all insecticides ^3^. Several mechanisms, including metabolic detoxification, target site mutations and cuticular genes are responsible for insecticide resistance ^4, 5^. Most common mechanisms associated with insecticide resistance in *Anopheles gambiae* s.l. include target-site resistance, notably knockdown resistance (kdr mutation with the 1014F and 1014S alleles responsible for resistance to DDT and pyrethroids) and the acetylcholinesterase (Ace-1) G119S mutation responsible for resistance to organophosphates and carbamates ^5– 7^. Metabolic resistance is another major resistance mechanism, occurring through the upregulation of several detoxification genes from three main families, the esterases, cytochrome P450 monooxygenases, and glutathione S-transferases responsible for resistance to different insecticide families and pollutants ^4^. There is a growing concern about the negative impact that insecticide resistance could have on malaria control. It increases the survival rate of mosquitoes exposed to insecticides in treated areas which could potentially lead to greater population size, increase in mosquito burden and diseases transmission ^8, 9^. Studies conducted so far suggested that resistant alleles could be associated with negative pleiotropic effects that could affect mosquito fitness, vectorial competency and disrupts the normal physiological functions of the mosquito ^10, 11^. Insecticide resistance has always been associated with lower fecundity, longer developmental time, reduced longevity, and lower mating success ^12, 13^. With the increasing use of insecticides, mosquitoes have been reported over recent years to have become multiresistant to different insecticide compounds ^14– 17^. Understanding the influence of insecticide resistance on vector population dynamic is becoming crucial for the implementation of insecticide resistance management strategies. Although resistance is largely expanding in *An. gambiae* s.l. populations from Cameroon, there has been so far little information on the influence of pyrethroid resistance on *An. gambiae* s.l. fitness. Experimental infection studies comparing resistant *versus* susceptible colonies have suggested increased prevalence of *Plasmodium* infections in *An. gambiae* s.s. resistant strains ^18– 20^. Studies on the malaria vector *An. funestus* s.s. indicated that the presence of the L119F-GSTe2 resistant allele was associated with reduced fecundity, increased larval developmental time and adult longevity ^21^. Further analysis suggested that this mechanism could also influence the vectorial capacity of resistant *An. funestus* s.s. populations ^22^. During the last decade important changes have been reported in *An. gambiae* s.l. populations from the city of Yaoundé with populations becoming increasingly tolerant to organic pollution ^23^, more resistant to pyrethroids and to different compounds ^14, 24, 25^, changes in the biting behavior was also reported ^26^. Yet the influence of these changes on the vectorial capacity or the fitness of *An. gambiae* s.l. populations has not been fully addressed.

In the present study an insecticide resistant *An. coluzzii* colony from the city of Yaoundé, was compared to a susceptible *An. coluzzii* laboratory colony “the Ngousso colony” to determine life-traits parameters affected by the increased expansion of insecticide resistance in this vector population.

## Methods

### Study site

The study was conducted in the city of Yaoundé, the capital of Cameroon (3° 52’ 12 N; 11° 31’ 12 E) from September 2018 to September 2019. In Yaoundé, *An. gambiae* s.l. is the main malaria vector. In order to obtained representative sample of the resistant *An. gambiae* s.l. population from the city of Yaoundé, anopheline larvae used to build the colony, were collected from different districts and locations (Tsinga, Nsam, Nkolbisson, Obobogo, Mvog-beti, Nouvelle Route Bastos, Nouvelle Route Tam-Tam, and Nouvelle Route Nkoldongo). In Yaoundé, malaria is highly prevalent, with the transmission rate varying from 0 to 90 infected bites/man/year ^26^. LLINs is the main method used by the population to prevent from malaria transmission ^27^. According to recent record it is estimated that over 75% of households in Yaoundé own at least a net ^28^. Urban agriculture is also practiced on a large scale in the city and large quantities of pesticides are used by farmers and these, alongside the use of LLINs, are the main sources of insecticide selection for mosquitoes in the city ^29, 30^.

### Susceptible strain

The laboratory colony used in the study is the Ngousso strain, originating from the district of Ngousso in Yaoundé, reared since 2006. This Ngousso *An. coluzzii* strain is fully susceptible to both permethrin and deltamethrin (mortality rate: ≥98% after one hour exposition to WHO impregnated papers).

### Susceptibility assays and establishment of a resistant laboratory colony

Anopheline larvae were collected in standing water collections on the field. Once in the laboratory, larvae were pooled and reared at mean temperature of 30°C and 73–75% relative humidity. After emergence, males were separated from females and adult females aged 3 to 5 days were used to conduct bioassays with deltamethrin (0.05%), permethrin (0.75%), DDT (4%), bendiocarb (0.1%) and Malathion (5%) according to WHO guidelines ^13^.

The resistant *An. coluzzii* colony was established by regular selection (once every two generations) of mosquitoes, exposing 3 to 5 day-old unfed females and males to 0.05% deltamethrin for 12 generations. Batches of 20 to 25 mosquitoes per tube were exposed to 0.05% deltamethrin-impregnated papers for 1 hour. Bioassays were conducted at temperatures of 25±2°C and 70–80% relative humidity. Control tests were conducted using untreated papers. 24 hours after exposure, survivor male and female were pooled for the mating and fed with a 5% glucose solution. After selection, the susceptibility status of the F12 generation was checked for the following insecticides: permethrin (0.75%), DDT (4%), bendiocarb (0.1%), malathion (5%) and PBO (4%) in order to check the implication of P450 metabolic-based mechanisms.

## Isofemale rearing and life-trait assessment

### Blood meal

To ensure that mosquitoes would feed, the glucose solution was removed 24 hours before blood feeding. Anopheline aged 3 to 6 days were placed into three cages (30×30×30 cm) of 100 females each for each strain and blood fed for 20 minutes on an anaesthetized rabbit. After blood feeding, females were provided with glucose solution, to allow maturation of eggs. The engorgement rate was assessed by counting well fed females.

The study was conducted under the ethical clearance N° 2016/11/832/CE/CNERSH/SP delivered by the Cameroon National Ethics (CNE) Committee for Research on Human Health Ref D30-172/L/MINSANTE/SG/DROS/TMC of 04 April 2017.

### Fecundity and fertility

For each strain (resistant and susceptible), 100 gravid females were placed in individual cups with damp filter paper to enable them lay eggs. After oviposition, the number of eggs laid per female was counted under a stereo microscope and eggs batch from each female were placed in water (plastic basins (17×12×6.50 cm) containing 200 ml of fresh water) 24 hours after the day of oviposition. All females that laid eggs were counted and stored at -20°C into numbered eppendorf tubes containing desiccant for further analyses. The egg from each isofemale line was reared separately.

### Larval development

To reduce competition, a maximum of 50 larvae were reared per tray. Additional trays were used for females with more than 50 larvae. Larvae were fed using baby fish food (TetraMin) under standard insectary conditions. During the rearing process, water from the tray was replaced every two days to reduce the influence of evaporation or pollution. At the pupal stage, the number of pupae was recorded every day and they were transferred into paper cup (8.50×10×7cm), up to 30 pupae were placed per cup. Different information were recorded from each colony reared, the number of individual per larval stage, the length of larval development, the number of larvae reaching the pupae stage, the number of pupae emerging as adults and the sex ratio.

### Adult longevity

After emergence, the number of males and females was recorded in each cup. Adult mosquitoes were fed with a 5% glucose solution. Each female progeny was placed in separate cup and followed. The survival rate was assessed by recording the number of males and females dying each day in each cup. Dead mosquitoes were removed from each cup daily for each family and kept at -20°C in 1.5-ml Eppendorf tubes containing desiccant.

### Molecular identification

Genomic DNA was extracted from whole mosquitoes using a previously described protocol ^31^. Molecular identification analyses were performed following the SINE200 PCR method describeb by Santolamazza *et al.* ^32^ to identify species of the *An. gambiae* complex.

### Detection of knockdown resistance ( *kdr*)

The presence of the *kdr* allele was checked in field collected mosquitoes (F0) and the laboratory resistant colony (F12 generation). A set of 119 females of the F0 generation and 111 F12 generation were randomly selected in each group for *kdr* analysis. L1014F and L1014S mutations were screened using the Mx3005P Real-Time PCR System, as describe previously ^33^. The primers *kdr*-Forward (5’-CAT TTT TCT TGG CCA CTG TAG TGA T-3’) and *kdr*-Reverse (5’-CGA TCT TGG TCC ATG TTA ATT TGC A-3’) were used. The probes *kdr*W (5’-ACG ACA AAA TTT C-3’) and *kdr*E (5’-ACG ACT GAA TTT C-3’) labelled with fluorochrome FAM, were used to detect the mutant alleles (1014F and 1014S) while the probe Wildtype (5’-CTT ACG ACT AAA TTT C-3’) labelled with fluorochrome HEX, were used for the wild type susceptible alleles detection. The Master Mix solution (9 µl) contained 5 µl of Sensimix (Biogen), 0.13 µl of each probe coupled to allelic specific primer and 3.88 µl of water. The thermocycler was set to run samples following the temperature cycling condition of: 1 hold of 10 minutes at 95°C for initial denaturation, followed by 40 cycles each of 95°C for 10 seconds, and 60°C for 45 seconds. For each experiment, there was one homozygote resistant for L1014S and L1014F *kdr*; one heterozygote for L1014S and L1014F *kdr*; and one susceptible L1014L used as positive controls. The negative controls were wells with 1 μl of ddH _2_O.

### Statistical analysis

Statistical analyses were performed with the software Excel 2010 and the statistical analysis software R (version 4.0.0) via RCommander (Rcmdr Version 2.6-2) and RStudio (version 1.2.5042). The Shapiro-Wilk test was applied to assess whether eggs and larvae counts conform to a normal distribution. Comparison of proportions between the two strains was conducted using a chi-square (χ ^2^) test. Female fecundity and fertility was assessed by comparing respectively the oviposition and the hatching rate between both strains using Welch’s two-sample t-test. The life-trait parameters such as duration of larval development and longevity were assessed by comparing means of both susceptible and resistant strains using a Kruskal-Wallis non-parametric test. While those such as pupation, emergence, sex-ratio were assessed by comparing means of both strains using Welch’s two sample t-test. To draw survival curves or Kaplan-Meier curve of larvae and adults, we used the package ‘ survminer’ version 0.4.6 that contains the function ‘ggsurvplot ()’. The latest allow drawing curve with the ‘number at risk’ table and the ‘censoring count plot’. The level of significance of each test was set at α < 0.05. Scatter plots were obtained from genotypes scored using the MxPro-MX3005P qPCR Software and the fluorescence (ΔR) threshold adjusted manually for each dye, if necessary, to enable the correct scoring of positive controls. The allele frequency of individuals carrying the *kdr* mutation was calculated using the formula f(R) = (2 × RR + RS)/2N, with RR = total number of homozygote resistant; RS = total number heterozygote resistant; N = total number of mosquitoes screened for the *kdr* mutation. Genotype frequency was calculated as relative frequencies of the homozygote resistant and heterozygote resistant individuals.

## Results

### Blood feeding rate

In the susceptible colony, 93% of females successfully blood fed (280/300), while only 34% females successfully blood fed in the resistant colony (102/300), revealing a significant difference in blood feeding between the two colonies (χ ^2^= 147.68, df = 1, P <0.0001) ( Table 1).

**Table 1.** Comparison of the blood feeding rate, fecundity and fertility rate of susceptible vs resistant mosquitoes.

	Susceptible strain		Resistant strain		
Parameters	n/N	Rates (%)	n/N	Rates (%)	P-value
Blood feeding	280/300	93.33 *	102/300	34 *	p <0.0001
Laying	86/100	86 *	40/100	40 *	p <0.0001
Hatching rate	7101/9604 (larvae/eggs)	73.94 *	2459/3736 (larvae/eggs)	65.82 *	p <0.0001

Abbreviations: N: total number of mosquitoes initially allowed to blood feed and lay eggs; n: number of mosquitoes who successfully blood fed and laid eggs; * significantly different (P<0.05).

### Fecundity and fertility

After choosing 100 fully blood fed females of each strain for individual egg laying, in the susceptible group, 86 females laid. A total of 9604 eggs, corresponding to a mean of 111.67±5.36 eggs/female was recorded ( Table 2). In the resistant colony, 40 females laid 3736 eggs ( Table 1), which correspond to an average of 93.33±10.77 eggs/female for females which were able to lay ( Table 2). In the resistant and the susceptible colonies, eggs count was found in conformity with a normal distribution Shapiro-Wilk normality test (resistant: W = 0.95, P = 0.06, susceptible: W = 0.97, P = 0.05).

**Table 2.** Differences in life-traits parameters between susceptible and resistant *Anopheles coluzzii*.

	Susceptible strain		Resistant strain		chi-squared & t test	P-value
Parameters	N	Means ± SE	N	Means ± SE		
Eggs	9604	111.67±5.36*	3736	93.33±10.77*	42.86	p <0.0001
Larvae	7101	83.54±4.76*	2459	63.05±9.49*	2.78	0.004
Larval development time (days)	-	7.57±0.18*	-	10.61±0.17*	-	0.035
Pupae	5434	63.19±4.37	1976	49.40±7.7	-1.18	0.12
Emergence	5226	54.26±6.76	1907	47.68±7.48	-0.33	0.74
Dead	238	2.7±0.39	61	1.53±0.29	1.26	0.21
Males	2530	26.43±3.31	982	24.55±3.78	-2.89	0.15
Females	2696	28.10±3.53*	925	23.13±3.75*	3.10	0.032
Adult live span	Male	20.77±0.52*	-	15.35±0.41*	1696	p <0.0001
	Female	21.71±0.47*	-	13.90±0.46*	4350	p <0.0001

Means±standard error is shown in all cases. Numbers followed by the star differ significantly (P<0.05); N: number.

Comparison between the two groups resistant vs susceptible indicated that the average number of eggs laid by resistant females was statistically lower (Kruskal-Wallis χ ^2^= 42.86, df = 1, P = 5.89×10 ^-11^). Fecundity parameters recorded were significantly higher in the susceptible colony compare to the resistant colony (χ ^2^ = 43.44, df = 1, P = 2.19×10 ^-11^) ( Table 2). The mean number of larvae was statistically lower in the resistant colony compare to the susceptible ( Table 2) (Welch Two Sample t-test: t = 2.78, df = 53, P = 0.004).

### Larval and pupae development

**Figure 1.**
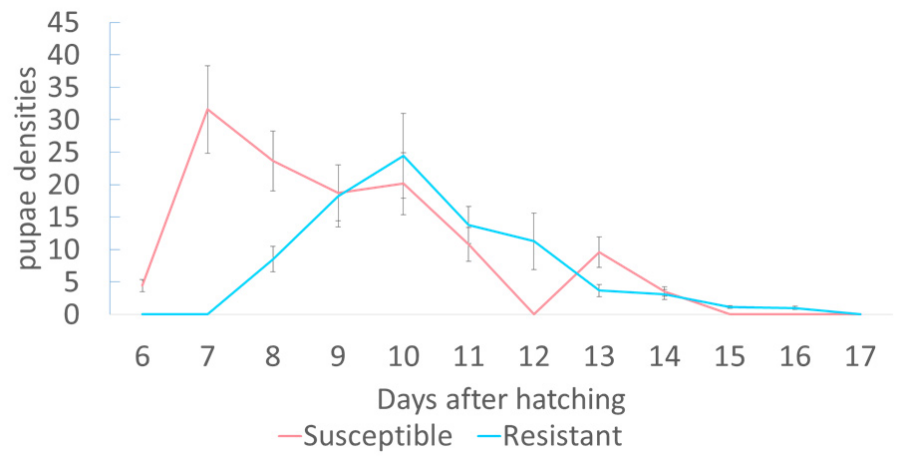
Mean of larval development time of susceptible and resistant larvae. Error bars = standard deviation.

The average length of larval development from hatching to pupation was compared between the two colonies. The average length of larval development to the pupa stage was 7.57 days for the susceptible and 10.61 days for the resistant group ( Figure 1). Survival probability analysis indicated that, resistant larvae take much more time to arrive to the pupa stage compared to the susceptible (χ ^2^ = 2251, df = 1, P = <2×10 ^-16^). In the susceptible group, high pupation rate was recorded between days 7 and 8, whereas for resistant larvae a peak of pupation was detected on day 10 ( Figure 1).

### Mean number of pupae and emergence rate in susceptible and resistant strain

In the susceptible colony, out of 7101 first instars larvae, 5434 larvae successfully arrived at the pupae stage and 5226 emerged as adults. The following correspond to 76.52% getting to the pupae stage and 73.59% to the adult stage. Within resistant out of 2459 larvae of the first instar, 1976 successfully arrived at the pupa stage (80.36%) and 1907 (77.55%) emerged as adults. The average number of offspring pupae per female was not significantly different between the two groups (Welch two-sample t-test: t = -1.18, df = 95.16, P = 0.12) ( Table 2). The average number of adults emerging was also not significantly different between the two groups (Welch two-sample t-test: t = -0.67, df = 82.33, P = 0.25) ( Table 2). Mortality during emergence was similar between susceptible and resistant groups (Welch two-sample t-test: t = 1.26, df = 54.34, P = 0.10) ( Table 2).

### Sex-ratio of adults mosquitoes

Of the 5226 mosquitoes who successfully emerged as adults in the susceptible group, 48.41% (N=2530) were males and 51.59% (N=2696) were females. Of the 1907 mosquitoes who successfully emerged as adults in the resistant group, there was 51.49% (N=982) of males and 48.51% (N=925) females. The proportion of emerging female was statistically similar to that of male in each offspring group, susceptible colony (χ ^2^ = 0.17, df = 1, P = 0.68) and resistant colony (χ ^2^ = 0.06, df = 1, P = 0.81). There were significantly more susceptible female than resistant female in the offspring (Welch two-sample t-test: t = 3.10, df = 61.99, P = 0.001) ( Table 2).

### Life span of the progeny of susceptible and resistant adults mosquitoes

**Figure 2.**
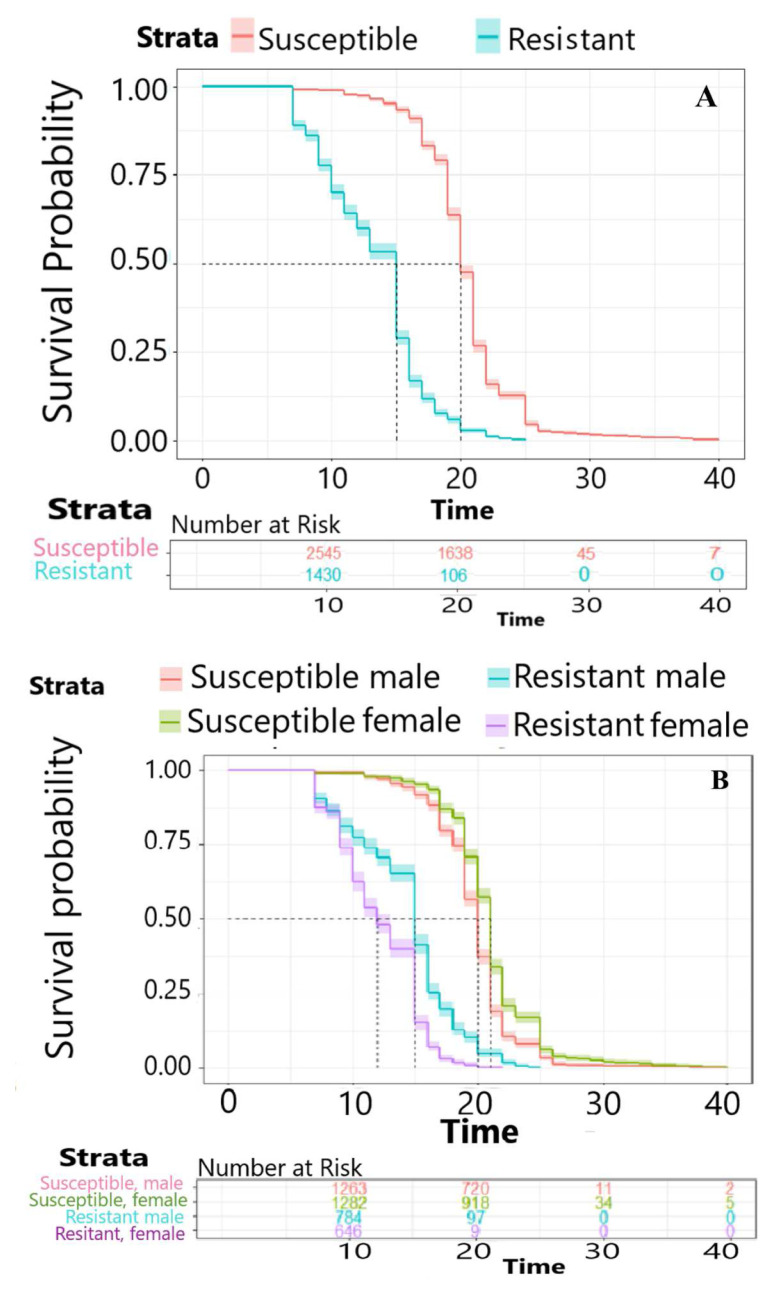
Life span and survival probabilities of mosquitoes. Comparison of the adult life span of susceptible and resistant mosquitoes ( **A**); Survival probability of male and female of susceptible and resistant mosquitoes ( **B**). X-axis = time in days, Y-axis = probability of surviving. Lines = survival curves of differents groups (strata). Vertical tick mark = half live time of each group.

The life span after emergence of the progeny of resistant and susceptible mosquitoes was assessed. Susceptible individuals appeared to live longer than resistant individuals ( Figure 2A) (χ ^2^ = 4350, df = 1, P = <2×10 ^-16^). The average life span for susceptible individuals was similar between males (20.76±0.52 days) and females (21.71±0.47 days), (t = -1.33, df = 81, p = 0.18). In the resistant group, females had a significantly shorter life span (13.90±0.46 days) than males (15.35±0.41 days), (t = 2.35, df = 30, p = 0.02), ( Table 2). The difference between the average life span number of males and females in each group was not significant for the susceptible group (t=2.58, df = 81, p = 0.9) whereas it was significant for the resistant group (t = 2.75, df = 30, p = 0.005). ( Figure 2B).

### Insecticide resistance profile of F0 and F12 generation

**Figure 3.**
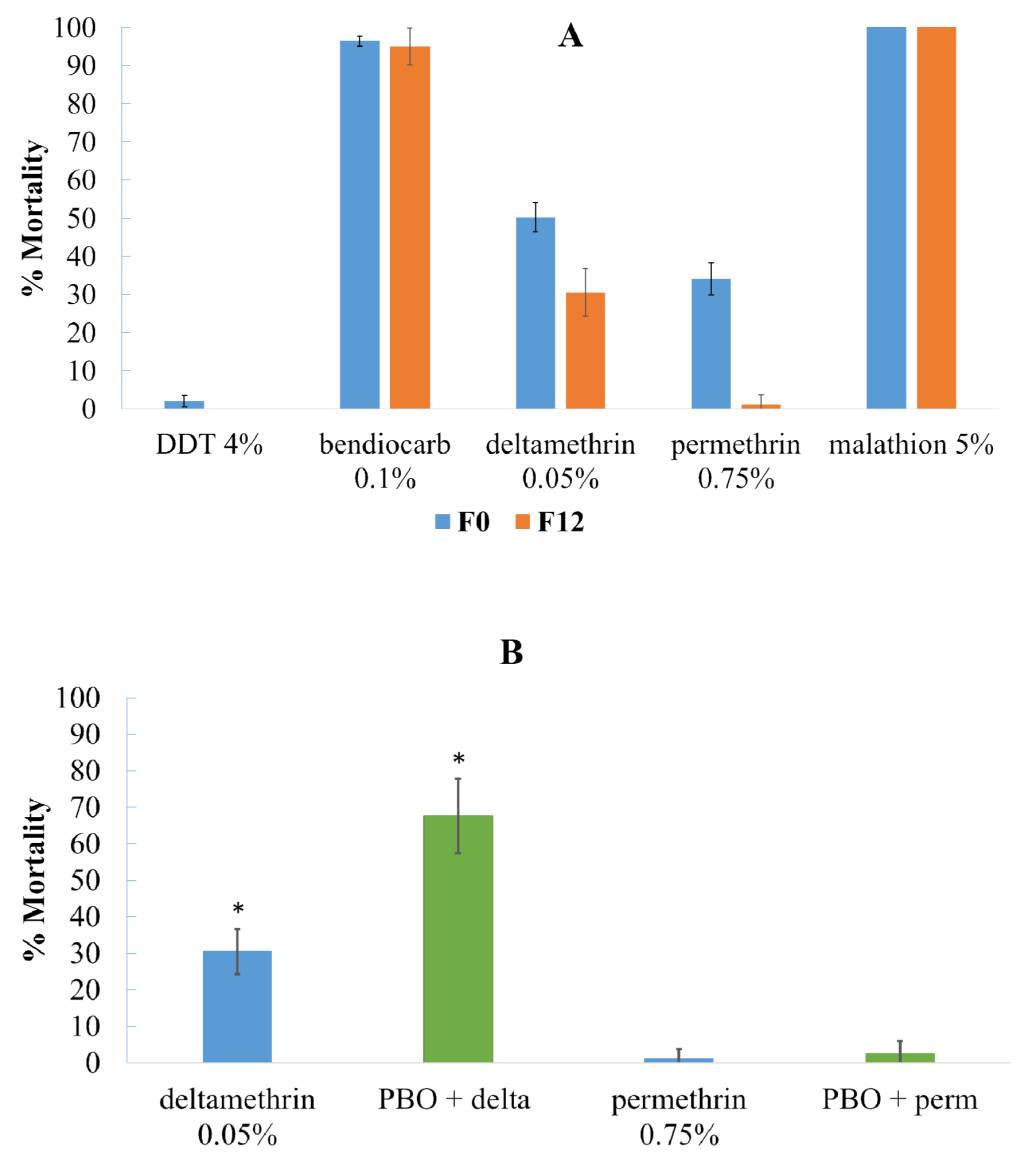
Mortality rates following insecticide exposure. Mortality of F0 and F12 generation to different insecticides ( **A**). Susceptibility status of F12 generation after preexposition to PBO ( **B**). * P<0.05, PBO+delta=PBO 4% + deltamethrin 0.05%, PBO+perm=PBO 4% + permethrin 0.75%. Error bars= 95% confidence intervals.

Field-collected mosquitoes from the F0 generation had a mortality rate of 2.05% [0.55-3.56] for DDT, 32.16% [29.94-38.37] for permethrin, 50.23% [46.37-54.10] for deltamethrin, 96.42 [96.38-96.48] for bendiocarb and 100% for malathion. The high resistance status of the strain to deltamethrin was maintained through generations, with the mortality rate decreasing from 50.23±3.86% for the F0 generation to 30.48±6.23% for the F12 generation. In the same way, the F12 generation showed 0% mortality to DDT 4%, 1.25% mortality rate to permethrin 0.75%, no change in their susceptibility to bendiocarb 0.1% (mortality rate: 95±1.35%) and malathion 5% (mortality rate: 100%) ( Figure 3A). When mosquitoes of the F12 generation were preexposed to PBO a mortality rate of 67.50±6.87% to deltamethrin 0.05% was recorded whereas, no variation in the mortality to permethrin 0.75% was recorded. ( Figure 3B).

### Molecular identification and *kdr* detection

Molecular identification performed on field-collected mosquitoes (F0 generation) showed that 7.53% (7/93) belong to *An. gambiae* and 92.47% (86/93) were *An. coluzzii*. While identification of 103 susceptibles and 311 resistants (F12 generation) mosquitoes confirms that the two groups belong to *An. coluzzii* species. The *kdr* allele 1014F was detected both in the field collected and the resistant group. The field-collected mosquitoes (F0) revealed 24/119 were homozygotes resistant, 90/119 were herterozygotes and 5/119 were homozygotes susceptibles, while in the F12 generation, all 111 individuals had the *kdr* West allele 1014F to make them homozygote resistant. The frequency of the L1014F *kdr* mutation was 58% for the F0 generation and 100% for the F12 generation. The 1014S allele was not detected ( Table 3).

**Table 3.** Genotypes and alleles frequencies of 1014F kdr mutation in resistant *An. coluzzii* populations.

	Genotypes						Alleles	
	RR		RS		SS			
Generation	n/N	%[95%CI]	n/N	%[95%CI]	n/N	%[95%CI]	2N	f(R)
F0	24/119	21.17	90/119	75.63	5/119	4.20	238	0.58
		[14.29-29.11]		[67.92-83.34]		[0.56-7.80]		
F12	111/111	100	0		0		222	1
P-value		P < 0.0001						

Abbreviations: RR: homozygous resistant; RS: heterozygous 1014F; SS: homozygous susceptible f (): frequency of the allele; [95%CI]: 95% confidence interval; N: total number of mosquitoes initially processed; n: number of mosquitoes successfully screened for the *kdr* mutation; F0: field collected population ; F12: population selected to deltamethrin 0.05% for 12 generations.

Raw data for this study are available, see *Underlying data* ^34^.

## Discussion

Insecticide resistance is rapidly expanding in *An. gambiae* s.l. population from the city of Yaoundé ^14, 35^. Yet the influence of insecticide resistance on *An. gambiae* s.l. species life trait and evolution is not well understood ^36^. The present study was undertaken to assess the influence of insecticide resistance on the fitness and life trait of *An. coluzzii* population by comparing a susceptible to a resistant colony. The study indicated high fitness cost in the resistant compared to the susceptible colony. This was consistent with previous observations done on the vector *An. funestus* s.s. in Cameroon ^11, 20^. Mosquitoes in the city of Yaoundé have been reported to be resistant to pyrethroids, and also to a large set of compounds including DDT, carbamate and pollutants ^24, 30, 35, 37^. Comparison of many life-trait parameters between the two colonies indicated differences at different levels. The blood feeding success was high in susceptible compare to resistant. The difference could have resulted from the fact that the susceptible colony was more adapted to blood feeding on rabbit blood as it has been maintained in laboratory for almost 14 years, whereas this is not the case for the resistant colony which has just been colonized in the insectary. Similar observations have been reported elsewhere ^12^. According to Martins *et al*., the blood meal is a key parameter which directly affects the general fitness, since it influences the number of eggs laid ^12^. Eggs from susceptible *An. coluzzii* also showed a high hatching rate and viability compared to those from resistant *An. coluzzii*. The results somewhere suggest that the rate of insemination could be lower in the resistant strain compared to the susceptible. Fecundity and fertility were also found to be reduced in resistant *An. funestus* ^21^, it is likely that this could be associated with resistance phenotype. Briegel *et al*. demonstrated that fecundity increases with successive blood meal, for *An. gambiae* s.l. it increases to 50% when two blood meals are provided for a single gonotrophic cycle ^38^.

The length of larval development in the resistant colony was three days longer than that of susceptible, similar findings have been reported for *An. funestus* s.s. ^21^. The longer the larval development time suppose higher exposition to pollutants and physico-chemical parameters from the breeding sites and more vulnerability to natural predators. A shorter development time is likely to accelerate adult emergence, leading to increase vector density, which is an important parameter of the vectorial capacity. In previous laboratories studies, Grimnig *et al*. suggested that extended larval development time for *An. gambiae* s.l. could result from high larval densities ^39^, but such a hypothesis is excluded here because the density of larvae per tray was below 50, which is sufficient for a good growth. The longer development time likely translates the influence of insecticide resistance on the general metabolism of resistant mosquitoes. Increased deleterious effects on the development, associated to pyrethroid resistance, have been demonstrated in *Aedes aegypti* ^12, 40^. Tchouakui *et al*. also reported a longer developmental time for *An. funestus* s.s. larvae carrying the 119F-GSTe2 and the CYP6P9a-R resistant allele compared to those with susceptible allele in Cameroon ^11, 21^. The expression of resistance genes has also been reported to induce alteration of some functions such as larval motility which could alter their capacity to look for food ^41, 42^

The density of pupae and the transition to adult stage were not significantly different between the two strains, suggesting that fitness cost mostly affects the larval stage and has no visible effect on pupae and adult emergence. The following could derive from the fact that pupae do not feed and there is limited influence of exogenic factors such as density or nutrient on this stage ^39, 43, 44^. No significant distortion of the sex ratio was recorded neither in the susceptible nor in the resistant colony. The absence of difference at this level, particularly for the resistant colony, could derive from the reduce number of larvae succeeding at the pupae and adult stage. In the nature, one male can inseminate several females during its lifespan, while females just need a unique insemination to accomplish multiple gonotrophic cycle for the rest of their life ^45^. Therefore, the number of females reaching the adult stage is an important parameter of reproduction success and vectorial capacity whereas this is not true for males ^45^.

Despite a similar life span between males and females in each colony, the progeny of susceptible mosquitoes was found to live longer in general 6 to 7 days longer than resistant mosquitoes. To be able to transmit malaria parasites it is important that the mosquitoes live long enough to enable the extrinsic development of the parasite in the mosquito which last approximately 12 days. From the study, it clearly appeared that if resistant mosquitoes are not infected after their first blood meal, few are going to be involved in malaria transmission since the average life span of resistant mosquitoes is estimated to be about 14 days for females. Vectors longevity is considered as a key factor contributing to the vectorial capacity of mosquitoes in endemic settings ^18, 21^. It is possible that the reduced life span of resistant *An. gambiae* s.l. mosquitoes in Yaoundé is negatively influencing its vectorial capacity. Reduced vectorial capacity associated with shorter longevity has previously been reported for pyrethroid-resistant *Aedes aegypti* populations in Brazil and in Thailand ^12, 40, 46^. As oppose to these findings, increased longevity, an increased vectorial competency was reported for F1 resistant *An. funestus* s.s. possessing the 119F-GSTe2 allele ^21, 47^. It is possible that in the present situation, the resistance status of *An. coluzzii* to insecticides also affects its vectorial competence. These findings still need to be validated by extensive field studies and experiments. Although the use of pyrethroid treated nets are considered to induce a low mortality in resistant individuals, resistant mosquitoes were however found to exhibit reduce blood feeding rate, low fecundity and short adult survival rate all this somewhere suggest a long term impact of insecticide base intervention (such as pyrethroid treated nets) selection on vector population. This long term negative impact confirm continuous performance of pyrethroid treated nets interventions on vector populations. This unrecognized impact of treated nets need to be highlighted in different epidemiological settings. Out of the two species *An. gambiae* and *An. coluzzii* identified in F0 generation, only *An. coluzzii* has successfully been maintained in laboratory across generations. The disappearance of the *An. gambiae* species could be explained by the low number of specimens and probably to the low adaptation capacity of the species to laboratory conditions. Molecular analysis of the resistant colony suggested the exclusive presence of the *kdr* West allele (1014F) at a high rate in the resistant colony. As mentioned in previous studies, the *kdr* mutation alongside metabolic detoxification could be the main mechanisms involved in pyrethroid resistance in *An. coluzzii* from Yaoundé ^16, 48, 49^. Detoxification genes such as *Cyp6p3*, *Cyp6m2* and *Cyp9k1*, have been reported involved in pyrethroid resistance in *An. gambiae* s.s. and *An. coluzzii* populations from Yaoundé ^30, 35, 50^.

## Conclusion

The study suggests that increase expansion of insecticide resistance in *An. coluzzii* populations from the city of Yaoundé, is likely associated with accumulation of deleterious effects affecting the life-traits of *An. coluzzii*. It appears from the study that the longer development time could render resistant larvae more vulnerable to control measures such as larviciding and to predators. It also appeared that adult resistant mosquitoes are associated with reduce fecundity, blood feeding rate and short survival rate, all these could affect adult vectorial capacity. Data generated from the present study, could be used to improve vector control strategies to be implemented on the field.

## Data availability

### Underlying data

Open Science Framework: Nkahe *et al.* 2020. https://doi.org/10.17605/OSF.IO/C8EUX ^34^.

This project contains the following underlying data:

- Data on life-traits (XLSX). (Trait data from the mosquiotes captured during this study.)
- pcr leslie (XLSX). (PCR data from this study.)
- Data Dictionary (DOCX).

Data are available under the terms of the Creative Commons Zero "No rights reserved" data waiver (CC0 1.0 Public domain dedication).
