# Supplementary material for: Fitness cost of insecticide resistance on the life-traits of a Anopheles coluzzii population from the city of Yaoundé, Cameroon
Source: Wellcome Open Res. 2020 Sep 8;5:171. Originally published 2020 Jul 20. [Version 2] doi: 10.12688/wellcomeopenres.16039.2 (PMC7525343; doi:10.12688/wellcomeopenres.16039.2)
Supplement: Peer review history

# Peer review: Reviewer response for version 2

**DOI:** 10.21956/wellcomeopenres.17911.r40323

**Published:** 2020-09-29

**Authors:** Ako Victorien Constant Edi

**Affiliations:** 1 Centre Suisse de Recherches Scientifiques en Côte d’Ivoire, Abidjan, Cote d'Ivoire

The authors considered the comments and made the necessary corrections within the manuscript.

 I have no further comments to make.

Is the work clearly and accurately presented and does it cite the current literature?

Yes

If applicable, is the statistical analysis and its interpretation appropriate?

Yes

Are all the source data underlying the results available to ensure full reproducibility?

Yes

Is the study design appropriate and is the work technically sound?

Yes

Are the conclusions drawn adequately supported by the results?

Yes

Are sufficient details of methods and analysis provided to allow replication by others?

Yes

Reviewer Expertise:

Vector biology, medical entomology and molecular biology and Epidemiology

I confirm that I have read this submission and believe that I have an appropriate level of expertise to confirm that it is of an acceptable scientific standard.

# Peer review: Reviewer response for version 2

**DOI:** 10.21956/wellcomeopenres.17911.r40324

**Published:** 2020-09-22

**Authors:** Yaw A. Afrane

**Affiliations:** 1 Department of Medical Microbiology, College of Health Sciences, University of Ghana, Accra, Ghana

I still think this paper cannot be indexed because the life history traits of an insectary susceptible strain bred over 10 years cannot be compared with that of a colony from the field that has been selected with an insecticide for several generations. The comparison should have been made with another colony from the field which will not be selected by any insecticide that will lose the resistance status over time. Insectary strain that has been kept for over 10 years have different life history traits such as fecundity, development time of the larvae, pupation rates etc. compared to a strain selected with an insecticide every other generation. Several studies have shown that resistant mosquitoes have a fitness cost (Alout *et al.*, 2014 ^1^).

 Therefore, what do the authors want to show by comparing that with a lab susceptible strain?

 My issue with the paper is in the design of the experiment based on what I have raised even if "it doesn't make any sense" to the authors.

Is the work clearly and accurately presented and does it cite the current literature?

Partly

If applicable, is the statistical analysis and its interpretation appropriate?

Yes

Are all the source data underlying the results available to ensure full reproducibility?

Yes

Is the study design appropriate and is the work technically sound?

No

Are the conclusions drawn adequately supported by the results?

No

Are sufficient details of methods and analysis provided to allow replication by others?

Yes

Reviewer Expertise:

Mosquito ecology

I confirm that I have read this submission and believe that I have an appropriate level of expertise to state that I do not consider it to be of an acceptable scientific standard, for reasons outlined above.

## References

[1] : Insecticide exposure impacts vector-parasite interactions in insecticide-resistant malaria vectors. *Proc Biol Sci*.2014;281(1786) : 10.1098/rspb.2014.0389 10.1098/rspb.2014.0389 24850924PMC4046407

# Peer review: Reviewer response for version 1

**DOI:** 10.21956/wellcomeopenres.17594.r39620

**Published:** 2020-09-02

**Authors:** Yaw A. Afrane

**Affiliations:** 1 Department of Medical Microbiology, College of Health Sciences, University of Ghana, Accra, Ghana

The authors are reporting their study on the “Fitness cost of insecticide resistance on the life-traits of a *Anopheles coluzzii *population from the city of Yaoundé, Cameroon”. Insecticide resistance is the most important factor limiting the success of malaria control program in Africa. More data are needed to understand the effect of insecticide resistance on malaria mosquitoes; the fitness cost involved in getting resistance and how it affects their potential to transmit malaria and other vector-borne diseases.  Therefore, the authors have chosen an important subject in malaria research to undertake their study.

 However, enthusiasm is diminished by weaknesses in the design of the study, and to some extent, the presentation of the results, making it difficult to recommend it for publication.

 Insectary strains of mosquitoes are known to have reduced resilience compared to wild caught mosquitoes. Being kept in captivity over several generations over the years makes the mosquitoes lose so many life-history traits, including resistant alleles, more especially as they are not under any selection pressure. Wild mosquitoes are under several pressures that could affect their malaria transmission potential. For a colony that has been kept for 14 years since 2006, their life history traits have been affected considerably compared to mosquitoes in the wild. Therefore, to make a comparison of wild-caught resistant mosquitoes that have been selected with deltamethrin for 12 generations with insectary colonized ones is not sound in my opinion. There should have been another wild-caught colony mosquitoes from the same area that were not selected with insecticide which would eventually lose their resistant abilities at some point to make the comparison. This colony would have been the unselected or susceptible strain. The insectary colony should have been a control strain for this experiment.

 Because of this weakness in the design of the experiment, I am unable to recommend it for publication.

 Other comments are as follows:

- The manuscript needs a revision for its language to bring clarity to the text.

**Methods:**

- It is not clear if the same mosquitoes that were hatched from the blood feeding of the adults in the first instance, were the same mosquitoes that were reared to study their development time, pupation rate, emergence rate, and also for their adult longevity. If it is the same that was used then this should be clearly stated.

 

**Results**

- Under the heading “Fecundity and fertility” There is no information on the susceptible colony. Please compare the two colonies.
- Under the heading “Life span of the progeny of susceptible and resistant adults Mosquitoes”  What was the difference between resistant and susceptible colonies for males and females. This was compared
- Under the heading “Molecular identification and *kdr” It*he authors report that “molecular identification performed on field-collected mosquitoes
  (F0 generation) showed that 7.5% (7/93) belong to *An. gambiae *and 92.47% (86/93) were *An. coluzzii*.” They go on to state that the F12 generation was all *An. coluzzii. *What happened to the *An. gambiae *population over time? Did they become fewer, got eliminated or their numbers were increased but were not part of the species ID done at the 12 ^th^ generation.
  In addition, a better clarification of how many colonies involved in the study is needed in that section.  
- Figure 2 legend needs revising. What actually is “B”? There is also no explanation to the figure called “Strata” in both Fig 2A and 2B

Is the work clearly and accurately presented and does it cite the current literature?

Partly

If applicable, is the statistical analysis and its interpretation appropriate?

Yes

Are all the source data underlying the results available to ensure full reproducibility?

Yes

Is the study design appropriate and is the work technically sound?

No

Are the conclusions drawn adequately supported by the results?

No

Are sufficient details of methods and analysis provided to allow replication by others?

Yes

Reviewer Expertise:

Mosquito ecology

I confirm that I have read this submission and believe that I have an appropriate level of expertise to state that I do not consider it to be of an acceptable scientific standard, for reasons outlined above.

# Response

**Published:** 2020-09-04

**Authors:** Christophe Antonio-N'kondjio

**Affiliations:** OCEAC, Cameroon

**Reviewer 3**

**Query 1: **The authors are reporting their study on the “Fitness cost of insecticide resistance on the life-traits of a *Anopheles coluzzii *population from the city of Yaoundé, Cameroon”. Insecticide resistance is the most important factor limiting the success of malaria control program in Africa. More data are needed to understand the effect of insecticide resistance on malaria mosquitoes; the fitness cost involved in getting resistance and how it affects their potential to transmit malaria and other vector-borne diseases.  Therefore, the authors have chosen an important subject in malaria research to undertake their study.

However, enthusiasm is diminished by weaknesses in the design of the study, and to some extent, the presentation of the results, making it difficult to recommend it for publication.

Insectary strains of mosquitoes are known to have reduced resilience compared to wild caught mosquitoes. Being kept in captivity over several generations over the years makes the mosquitoes lose so many life-history traits, including resistant alleles, more especially as they are not under any selection pressure. Wild mosquitoes are under several pressures that could affect their malaria transmission potential. For a colony that has been kept for 14 years since 2006, their life history traits have been affected considerably compared to mosquitoes in the wild. Therefore, to make a comparison of wild-caught resistant mosquitoes that have been selected with deltamethrin for 12 generations with insectary colonized ones is not sound in my opinion. There should have been another wild-caught colony mosquitoes from the same area that were not selected with insecticide which would eventually lose their resistant abilities at some point to make the comparison. This colony would have been the unselected or susceptible strain. The insectary colony should have been a control strain for this experiment.

Because of this weakness in the design of the experiment, I am unable to recommend it for publication.

Other comments are as follows:

- The manuscript needs a revision for its language to bring clarity to the text.

**Response 1:** We used a laboratory strain as control because it was not possible to have a field susceptible population. With the expansion of insecticide resistance in mosquito populations it has now become difficult to find susceptible field population in Yaoundé. The Ngousso strain used in this case as control originate from Yaoundé and is of the species *An. coluzzii *and is known for its high susceptibility to all insecticide classes. Several experimental study design comparing laboratory strains to field population have been reported in the literature and are still conducted nowadays. For instance the Kisumu laboratory strain is largely used for experimental studies and also in WHO bioassays to check the quality of impregnated papers. The argument that using a laboratory strain is a major weakness in the design of our study doesn’t make sense we totally disagree with the reviewer's point of view.

**Method**

**Query 2 :** It is not clear if the same mosquitoes that were hatched from the blood feeding of the adults in the first instance, were the same mosquitoes that were reared to study their development time, pupation rate, emergence rate, and also for their adult longevity. If it is the same that was used then this should be clearly stated.

**Response 2:** The same mosquitoes were followed in the course of the study, please refer to the text and the raw data file for further details.

**Results**

**Query 3:** Under the heading “Fecundity and fertility” There is no information on the susceptible colony. Please compare the two colonies.

**Response 3: **The information is clearly presented in the section (please read through the section).

**Query 4:** Under the heading “Life span of the progeny of susceptible and resistant adults Mosquitoes” What was the difference between resistant and susceptible colonies for males and females. This was compared

**Response 4:** The information is provided in figure 2, please check graph B (and the legend above the graph for details).

**Query 5:** Under the heading “Molecular identification and *kdr” t*he authors report that molecular identification performed on field-collected mosquitoes (F0 generation) showed that 7.5% (7/93) belong to *An. gambiae *and 92.47% (86/93) were *An. coluzzii*.” They go on to state that the F12 generation was all *An. coluzzii. *What happened to the *An. gambiae *population over time? Did they become fewer, got eliminated or their numbers were increased but were not part of the species ID done at the 12 ^th^ generation.

In addition, a better clarification of how many colonies involved in the study is needed in that section.  

**Response 5:** PCR analysis of over 200 additional samples from the resistant colony (F12 generation) indicated that it was constituted of only *An. coluzzii* individuals.

**Query 6:** Figure 2 legend needs revising. What actually is “B”? There is also no explanation to the figure called “Strata” in both Fig 2A and 2B

**Response 6:** “B” refers to the second graph on the figure ( the survival probability of male and female of the resistant and susceptible colonies). We provided additional details on the figure legend.

# Peer review: Reviewer response for version 1

**DOI:** 10.21956/wellcomeopenres.17594.r39619

**Published:** 2020-08-21

**Authors:** Fredros O. Okumu

**Affiliations:** 1 Environmental Health and Ecological Sciences Department, Ifakara Health Institute, Ifakara, Tanzania

The work by Nkahe *et al.* addresses an important question on how pyrethroid resistance might impact several life cycle traits of malaria vectors, and how this may translate to performance of control measures.

 The writing is excellent, study methods properly described and the scientific rationale clearly stated.

 I found it particularly interesting that susceptible mosquitoes live so much longer than resistant ones (~22 days compared to ~15 days). Coupled with the reduced blood-feeding proportions, this could mean very high levels of impact from insecticidal-interventions even if they do not impart direct mortality. It would be greater if this was discussed further with respect to the expected performance of interventions such as pyrethroid-treated nets.

**Two minor comments are as follows:**

- In the discussion section, the authors indicate that most of the fitness costs were in the aquatic stages, yet we see some great reductions on fecundity and adult survival as well. Since vector survival has far greater impacts on pathogen transmission than actual densities, it is perhaps incorrect to conclude that the fitness costs are mostly in the pre-adult stages.
- Would be great to see clarity on the decision to use 12 generations of the resistant colonies

Is the work clearly and accurately presented and does it cite the current literature?

Yes

If applicable, is the statistical analysis and its interpretation appropriate?

Yes

Are all the source data underlying the results available to ensure full reproducibility?

Yes

Is the study design appropriate and is the work technically sound?

Yes

Are the conclusions drawn adequately supported by the results?

Yes

Are sufficient details of methods and analysis provided to allow replication by others?

Yes

Reviewer Expertise:

Public Health; Infectious Tropical Diseases; Vector Biology & Control

I confirm that I have read this submission and believe that I have an appropriate level of expertise to confirm that it is of an acceptable scientific standard.

# Response

**Published:** 2020-09-04

**Authors:** Christophe Antonio-N'kondjio

**Affiliations:** OCEAC, Cameroon

**Reviewer 2**

**info_outline**

The work by Nkahe *et al.* addresses an important question on how pyrethroid resistance might impact several life cycle traits of malaria vectors, and how this may translate to performance of control measures.

The writing is excellent, study methods properly described and the scientific rationale clearly stated.

**Query 1:** I found it particularly interesting that susceptible mosquitoes live so much longer than resistant ones (~22 days compared to ~15 days). Coupled with the reduced blood-feeding proportions, this could mean very high levels of impact from insecticidal-interventions even if they do not impact direct mortality. It would be greater if this was discussed further with respect to the expected performance of interventions such as pyrethroid-treated nets.

**Response 1: **The following phrase was added in the discussion section “Although the use of pyrethroid treated nets are considered to induce a low mortality in resistant individuals, resistant mosquitoes were however found to exhibit reduce blood feeding rate, low fecundity and short adult survival rate all this somewhere suggest a long term impact of insecticide base intervention (such as pyrethroid treated nets) selection on vector population. This long term negative impact confirm continuous performance of pyrethroid treated nets interventions on vector populations. This unrecognized impact of treated nets need to be evaluated in different epidemiological settings”.

**Two minor comments are as follows:**

**Query 2:** In the discussion section, the authors indicate that most of the fitness costs were in the aquatic stages, yet we see some great reductions on fecundity and adult survival as well. Since vector survival has far greater impacts on pathogen transmission than actual densities, it is perhaps incorrect to conclude that the fitness costs are mostly in the pre-adult stages.

**Response** **2: **The conclusion was corrected to address this comment.

**Query 3:** Would be great to see clarity on the decision to use 12 generations of the resistant colonies

**Response 3: **We selected mosquitoes for 12 generations to have highly resistant individuals and avoid susceptible individuals in the colony.

# Peer review: Reviewer response for version 1

**DOI:** 10.21956/wellcomeopenres.17594.r39850

**Published:** 2020-08-21

**Authors:** Ako Victorien Constant Edi

**Affiliations:** 1 Centre Suisse de Recherches Scientifiques en Côte d’Ivoire, Abidjan, Cote d'Ivoire

**General comments:**

- This study of Fitness cost of insecticide resistance on the life-traits of a *Anopheles coluzzii *population from the city of Yaoundé, Cameroon represents an important topic related to the insecticide resistance monitoring in the city of Yaoundé, Cameroon **. **
- Although resistance is largely expanding in *An. gambiae *s.l. populations from Cameroon, there has been little information so far on the influence of pyrethroid resistance on *An. gambiae *s.l. fitness and the current study generated data to support evidence.
- This study compared data from an insecticide resistant *An. coluzzii *colony from the city of Yaoundé, to a susceptible *An. coluzzii *laboratory colony “the Ngousso colony” to determine life-traits parameters affected by the increased expansion of insecticide resistance in this vector population.

**I recommend the current version of this paper for indexing with the very minor changes below to be considered by the authors.**

**Minor revisions:**

- Key-words: An. colluzzi to be put in italics ( *An. colluzzi*).

 

**Introduction section:**

- **In Line 3 to 5, **the sentence “ **Four insecticide families, organophosphates, organochlorines, carbamates and pyrethroids, are used in public health**” needs to be corrected. With the introduction of SumiShield (neonicotinoid Clothianidin) to the market, the number of classes of insecticides has increased.

 

**Methods section:**

**Study site: **

- Line 13 to 15: Please add the reference to the following sentence: “ **According to recent record it is estimated that over 75% of households in Yaoundé own at least a net**”.

**Results section:**

- Table 2. Differences in life-traits parameters between susceptible and resistant *Anopheles coluzzii*.
   
  - The Means ± SE of resistant strain should be 93.33±10.77* rather than 93.4±10.77*. Could you please check the writing of the numbers and choose if you want one or two numbers for the decimal and be constant throughout the manuscript.
- Sex-ratio of adults mosquitoes:
  - In the sentence: “Of the 5226 mosquitoes who successfully emerged as adults in the susceptible group, 48.41% (N=2530) were males and 51.6% (N=2696) were females.”, the sum of both percentages is 100.1% instead of 100%, therefore it is important to choose either to keep 1 or (48.4% and 51.6%) or two numbers for the decimal (48.41% and 51.59%). Check throughout the manuscript.
  - In the sentence: “Of the 1907 mosquitoes who successfully emerged as adults in the resistant group, there was 51.49% (N=982) of males and 48.50% (N=925) females.”, the percentage should be 48.51% to have 100% when sum up both percentage values. Check throughout the manuscript.
- Life span of the progeny of susceptible and resistant adults mosquitoes:
   
  - Line 10: In the sentence “The difference between the **average number** of males and females in each group was not significant for the susceptible group”, replace the average number by **average life span number.**
- Insecticide resistance profile of F0 and F12 generation:
   
  - What do the error bars mean on Figure 3 (confidence intervals or SEM?). Please add the meaning below the figure.
- Molecular identification and *kdr:*
   
  - Molecular identification performed on field-collected mosquitoes (F0 generation) showed that 7.5% (7/93) belong to *An. gambiae *and 92.47% (86/93) were *An. coluzzii*. While identification of 103 susceptible and 111 resistant (F12 generation) mosquitoes **confirms that the two groups belong to *An. coluzzii *species**. This finding is interesting and needs to be discussed in the Discussion section.
  - In Table 3: The sum of the percentage from N/119 is 101.53%, not 100%. This occurs because the percentage of RR should be 20.17% rather than 21.7%. Please check and correct throughout the manuscript.
  - In Table 3: The count of alleles (2N) should be 222 rather than 228 for the F12 generation ie. 2 x111. Please check and correct.
  - Please double check the number in each table and correct where necessary.

**Discussion section:**

- The authors with caution indicated that “ **It is possible that in the present situation, the resistance status of *An. coluzzii *to insecticides also affects its vectorial competence. These findings still need to be validated by extensive field studies and experiments**.” Validation by further experimental studies will be important to improve knowledge on relation between vector competency and longevity. 
- From F0, only *An. coluzzi *was selected over generation for female and male. Did the authors also find some *An. gambiae s.s* from emerging larvae across generations? If so what was the frequency relative to *An. coluzzii*?

**Level of interest: **An article of importance in its field.

**Statistics: **No statistical problem recorded.

**Quality of written English: **Acceptable.

Is the work clearly and accurately presented and does it cite the current literature?

Yes

If applicable, is the statistical analysis and its interpretation appropriate?

Yes

Are all the source data underlying the results available to ensure full reproducibility?

Yes

Is the study design appropriate and is the work technically sound?

Yes

Are the conclusions drawn adequately supported by the results?

Yes

Are sufficient details of methods and analysis provided to allow replication by others?

Yes

Reviewer Expertise:

Vector biology, medical entomology and molecular biology and Epidemiology

I confirm that I have read this submission and believe that I have an appropriate level of expertise to confirm that it is of an acceptable scientific standard, however I have significant reservations, as outlined above.

# Response

**Published:** 2020-09-04

**Authors:** Christophe Antonio-N'kondjio

**Affiliations:** OCEAC, Cameroon

**Reviewer 1**

**General comments:**

- This study of Fitness cost of insecticide resistance on the life-traits of a *Anopheles coluzzii *population from the city of Yaoundé, Cameroon represents an important topic related to the insecticide resistance monitoring in the city of Yaoundé, Cameroon **.** 
- Although resistance is largely expanding in *An. gambiae *s.l. populations from Cameroon, there has been little information so far on the influence of pyrethroid resistance on *An. gambiae *s.l. fitness and the current study generated data to support evidence. 
- This study compared data from an insecticide resistant *An. coluzzii *colony from the city of Yaoundé, to a susceptible *An. coluzzii *laboratory colony “the Ngousso colony” to determine life-traits parameters affected by the increased expansion of insecticide resistance in this vector population.

**I recommend the current version of this paper for indexing with the very minor changes below to be considered by the authors.**

**Minor revisions:**

**Query 1:** Key-words: An. coluzzii to be put in italics ( *An. coluzzii*).

**Response 1: **Done.

**Introduction section:**

**Query 2:** **In Line 3 to 5, **the sentence “ **Four insecticide families, organophosphates, organochlorines, carbamates and pyrethroids, are used in public health**” needs to be corrected. With the introduction of SumiShield (neonicotinoid Clothianidin) to the market, the number of classes of insecticides has increased.

**Response 2:** Information added, see introduction.

**Methods section:**

**Study site:**

**Query 3:** Line 13 to 15: Please add the reference to the following sentence: “ **According to recent record it is estimated that over 75% of households in Yaoundé own at least a net**”.

**Response 3:** Reference added.

**Results section:**

**Table 2.** Differences in life-traits parameters between susceptible and resistant *Anopheles coluzzii*.

**Query 4:** The Means ± SE of resistant strain should be 93.33±10.77* rather than 93.4±10.77*. Could you please check the writing of the numbers and choose if you want one or two numbers for the decimal and be constant throughout the manuscript.

**Response 4: **All changes done

**Sex-ratio of adult mosquitoes:**

**Query 5:** In the sentence: “Of the 5226 mosquitoes who successfully emerged as adults in the susceptible group, 48.41% (N=2530) were males and 51.59% (N=2696) were females.”, the sum of both percentages is 100.1% instead of 100%, therefore it is important to choose either to keep 1 or (48.4% and 51.6%) or two numbers for the decimal (48.41% and 51.59%). Check throughout the manuscript.

**Response 5: **Corrected and checking done throughout the manuscript.

**Life span of the progeny of susceptible and resistant adults mosquitoes:**

**Query 6:** Line 10: In the sentence “The difference between the **average number** of males and females in each group was not significant for the susceptible group”, replace the average number by **average life span number.**

**Response 6: **Changes done.

**Insecticide resistance profile of F0 and F12 generation:**

**Query 7:** What do the error bars mean on Figure 3 (confidence intervals or SEM?). Please add the meaning below the figure.

**Response 7:** The additional information was added below each figure

Figure 1: Error bars= Standard Deviation

Figure 3: *E*rror bars *= *confidence intervals

**Molecular identification and *kdr:***

**Query 8:** Molecular identification performed on field-collected mosquitoes (F0 generation) showed that 7.5% (7/93) belong to *An. gambiae *and 92.47% (86/93) were *An. coluzzii*. While identification of 103 susceptible and 111 resistant (F12 generation) mosquitoes **confirms that the two groups belong to *An. coluzzii *species**. This finding is interesting and needs to be discussed in the Discussion section.

**Response 8:** Only *An. coluzzii* was successfully maintained in laboratory across generations. The findings was discussed in the discussion section.

**Query 9:**

- In Table 3: The sum of the percentage from N/119 is 101.53%, not 100%. This occurs because the percentage of RR should be 20.17% rather than 21.7%. Please check and correct throughout the manuscript.
- In Table 3: The count of alleles (2N) should be 222 rather than 228 for the F12 generation ie. 2 x111. Please check and correct.

**Response 9:** Table 3 was corrected accordingly

**Query 10:** Please double check the number in each table and correct where necessary.

**Response 10:** Each number has been checked and corrected.

**Discussion section:**

- The authors with caution indicated that “ **It is possible that in the present situation, the resistance status of *An. coluzzii *to insecticides also affects its vectorial competence. These findings still need to be validated by extensive field studies and experiments**.” Validation by further experimental studies will be important to improve knowledge on relation between vector competency and longevity. 

**Query 11:** From F0, only *An. coluzzii *was selected over generation for female and male. Did the authors also find some *An. gambiae s.s* from emerging larvae across generations? If so what was the frequency relative to *An. coluzzii*?

**Response 11:** Apart from the f0 generation no *An. gambiae* larvae were found across generations. Over 200 additional specimens were processed and all turned to be *An. coluzzii*.
